# COVID-19 Response In U.S. Assisted Living Settings: Key Stakeholder Perspectives

**DOI:** 10.1093/geroni/igab046.219

**Published:** 2021-12-17

**Authors:** Sarah Dys, Jaclyn Winfree, Paula Carder, Sheryl Zimmerman, Kali Thomas

**Affiliations:** 1 Portland State University, Portland, Oregon, United States; 2 Institute on Aging, Portland State University, Portland, Oregon, United States; 3 OHSU-PSU School of Public Health, Portland, Oregon, United States; 4 Cecil G. Sheps Center for Health Services Research, Chapel Hill, North Carolina, United States; 5 Brown University, Brown University/Providence, Rhode Island, United States

## Abstract

Unique regulatory requirements and scope of services within assisted living (AL) pose distinctive challenges to COVID-19 response. To identify COVID-19 issues specific to AL, we recruited stakeholders with expertise in AL operations, policy, practice, and research (n=42) to participate in remote interviews between July and September 2020. Using thematic analysis, we derived the following overarching themes: 1) Policymakers lack an understanding of the AL context; 2) AL administrators were left to coordinate guidelines with little support; 3) AL organizations faced limited knowledge of and disparate access to resources; 4) State-level regulatory requirements conflicted with COVID-19 guidelines resulting in confusion; and 5) AL operators struggled to balance public health priorities with promoting their residents’ wellbeing. To develop evidence-informed policy and avoid unintended consequences, AL operators, direct care workers, residents, and clinicians practicing in these settings should have opportunities to provide feedback through the policy development process, both state and national.

